# Non-planar dielectrics derived thermal and electrostatic field inhomogeneity for boosted weather-adaptive energy harvesting

**DOI:** 10.1093/nsr/nwad186

**Published:** 2023-06-28

**Authors:** Yi Zhou, Tianpeng Ding, Yin Cheng, Yi Huang, Wu Wang, Jianmin Yang, Lin Xie, Ghim Wei Ho, Jiaqing He

**Affiliations:** Shenzhen Key Laboratory of Thermoelectric Materials and Department of Physics, Southern University of Science and Technology, Shenzhen 518055, China; Department of Electrical and Computer Engineering, National University of Singapore, Singapore 117581, Singapore; Department of Electrical and Computer Engineering, National University of Singapore, Singapore 117581, Singapore; School of Electronic Science and Engineering, State Key Laboratory of Electronic Thin Film and Integrated Devices, University of Electronic Science and Technology of China, Chengdu 611731, China; Department of Electrical and Computer Engineering, National University of Singapore, Singapore 117581, Singapore; Shenzhen Key Laboratory of Thermoelectric Materials and Department of Physics, Southern University of Science and Technology, Shenzhen 518055, China; Shenzhen Key Laboratory of Thermoelectric Materials and Department of Physics, Southern University of Science and Technology, Shenzhen 518055, China; Shenzhen Key Laboratory of Thermoelectric Materials and Department of Physics, Southern University of Science and Technology, Shenzhen 518055, China; Department of Materials Science and Engineering, National University of Singapore, Singapore 117575, Singapore; Shenzhen Key Laboratory of Thermoelectric Materials and Department of Physics, Southern University of Science and Technology, Shenzhen 518055, China; Department of Electrical and Computer Engineering, National University of Singapore, Singapore 117581, Singapore; Department of Materials Science and Engineering, National University of Singapore, Singapore 117575, Singapore; Institute of Materials Research and Engineering, Agency for Science, Technology and Research (A*STAR), Singapore 138634, Singapore; Shenzhen Key Laboratory of Thermoelectric Materials and Department of Physics, Southern University of Science and Technology, Shenzhen 518055, China; Guangdong-Hong Kong-Macao Joint Laboratory for Photonic-Thermal-Electrical Energy Materials and Devices, Southern University of Science and Technology, Shenzhen 518055, China

**Keywords:** non-planar dielectrics, heat manipulation, pyroelectrics, weather-adaptive, energy harvesting

## Abstract

Weather-adaptive energy harvesting of omnipresent waste heat and rain droplets, though promising in the field of environmental energy sustainability, is still far from practice due to its low electrical output owing to dielectric structure irrationality and unscalability. Here we present atypical upcycling of ambient heat and raindrop energy via an all-in-one non-planar energy harvester, simultaneously increasing solar pyroelectricity and droplet-based triboelectricity by two-fold, in contrast to conventional counterparts. The delivered non-planar dielectric with high transmittance confines the solar irradiance onto a focal hotspot, offering transverse thermal field propagation towards boosted inhomogeneous polarization with a generated power density of 6.1 mW m^−2^ at 0.2 sun. Moreover, the enlarged lateral surface area of curved architecture promotes droplet spreading/separation, thus travelling the electrostatic field towards increased triboelectricity. These enhanced pyroelectric and triboelectric outputs, upgraded with advanced manufacturing, demonstrate applicability in adaptive sustainable energy harvesting on sunny, cloudy, night, and rainy days. Our findings highlight a facile yet efficient strategy, not only for weather-adaptive environmental energy recovery but also in providing key insights for spatial thermal/electrostatic field manipulation in thermoelectrics and ferroelectrics.

## Teaser

Non-planar dielectrics are developed to manipulate thermal and electrostatic field inhomogeneities for weather-adaptive environmental energy harvesting with a two-fold increase in power output.

## INTRODUCTION

Harvesting weather-dependent non-static environmental energies, e.g. solar heat, wind energy, and raindrop energy, into electricity in order to maintain carbon sustainability/neutrality and economic growth, has been of interest for a few decades due to ever-increasing energy demand and urgent climate targets [1–5]. Ubiquitous environmental energy sources, such as solar illuminations, wind fluctuations, ground heat (superambient low-grade heat) and raindrops (kinetic energy), are typically season-/climate-dependent and present as sunny/cloudy/rainy weathers as well as diurnal cycles [1,2,6]. Although these disordered and decentralized energies are widespread, they are far less capitalized in comparison with the intensively exploited photovoltaics (PV) and steady-state/static spatial heat recovery [7–11], especially in weather-adaptive solar heat and raindrop energy harvesting. Recently, hybrid energy generators integrated with PV cells and triboelectrics have been demonstrated to scavenge solar and rain energies on sunny and rainy days individually. However, challenges remain for irradiance fluctuation which not only leads to inefficient photovoltaics but also results in unusable instantaneous heat variations. Explicitly, irradiance/weather changes make PV cells less stable for low-light intensity, or low-grade, non-static ambient heat harvesting, especially at night or in cloudy scenarios with wind-driven heat convection [12,13]. Moreover, the widely-used planar structure (e.g. PV cells, triboelectrics) with a low degree of freedom is not a priority to meet high-entropy energy harvesting of diffused, disordered, decentralized, and distributed energy sources due to its high demand for structural adaptiveness and device efficacy [14–17]. To circumvent these impediments, other energy harvesting alternatives driven by day/night environmental heat fluctuations and raindrops with co-designed dielectric structures are in urgent need of development for weather-adaptive energy recovery.

Pyroelectrics that generate electricity via temporal temperature change (d*T*/d*t*), show promise in scavenging the underexplored diffused environmental heat of unstable, non-static thermodynamic processes [18–21]. The pyroelectric effect is normally triggered by the dipole moment change in a unit volume of polar materials [22–26], in which the electrical output is governed by spontaneous polarization (*P*_S_) and non-static heat fluctuations. Recent studies have shown an increased output power density from 10 to 10^3^ μW m^−2^ for solar heat harvesting using pyroelectric energy harvesters [20,27–30]. Nonetheless, conventional pyroelectrics with a planar configuration capture the solar heat homogenously, limited by simultaneous and uniform thermal field propagation across the entire device (mechanistic see Fig. S1) [12,31,32]. These approaches with spatiotemporally coupled thermodynamic processes bring low d*T*/d*t* and temperature-dependent *P*_S_, thus restraining pyroelectric output [18,20]. Meanwhile, traditional raindrop energy harvesters mainly utilize flat triboelectric dielectrics with a tilting angle via electrostatic induction and triboelectrification during the liquid-solid contact/separation process [33–38]. There are a few, if any, studies on efficaciously harvesting solar/rain energies employing non-planar/curved bulk dielectrics with adaptiveness to attain inhomogeneous local heat and electrostatic field propagation towards increased pyro/triboelectric output.

**Figure 1.**
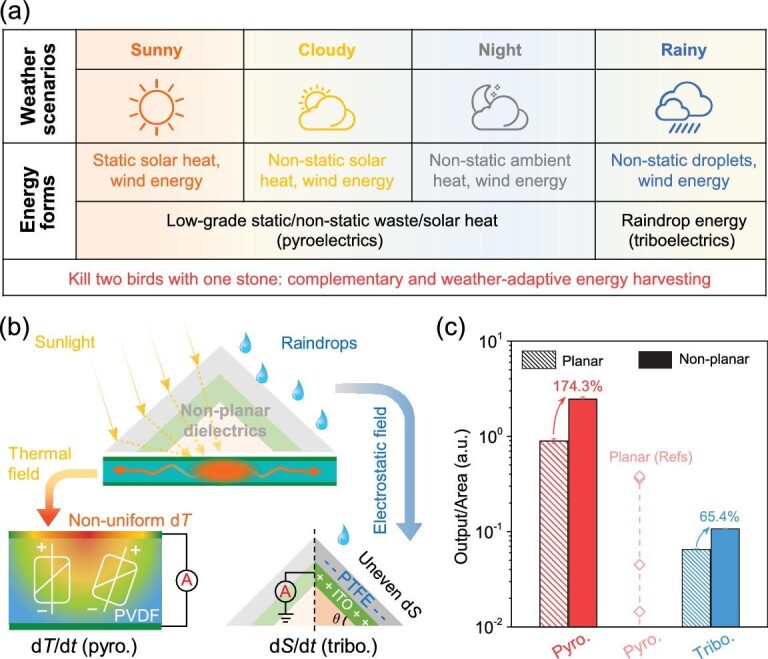
The conceptual mechanistic of weather-adaptive non-planar energy harvester. (a) Weather scenarios and corresponding energy quality for adaptive, complementary, and sustainable environmental energy harvesting. The weather-dependent heat and wind fluctuations induce pyroelectric polarization change towards electricity generation, e.g. solar heating, convection cooling, waste heat capture, and night condition (heat convection). In contrast, the falling rain droplet impinges on the dielectric and can offer electrostatic triboelectrification. (b) Mechanistic of non-planar dielectrics for confined thermal field inhomogeneity towards non-uniform temporal heat propagation and boosted solar pyroelectric (pyro.) heat harvesting (left), as well as uneven temporal droplet spreading area change to promote liquid-solid interfacial triboelectrification (tribo.) for droplet-based energy harvesting (right). (c) Normalized power output of non-planar energy generator versus planar for heat and rain harvesting. The power output of reported planar solar pyroelectrics is tabulated in Table S1.

In this work, we report controlled spatial thermal and electrostatic field inhomogeneity using unconventional non-planar dielectrics to ‘kill two birds with one stone’ towards overcoming the impediment of traditional environmental energy harvesters (Fig. 1, Note S1). Dissimilar from typical planar dielectrics, where homogeneous temperature and electrostatic field propagation are solely governed by the total input, our proposed non-planar device confines local solar heat propagation along the in-plane direction, thus increasing non-uniform d*T*/d*t* and *P*_S_ changes for enhanced pyroelectricity production. More interestingly, this non-planar dielectric with curved architecture and textured morphology promotes water droplet spreading/separation inhomogeneity, thereby boosting the overall electrostatic field towards enlarged triboelectricity (discussions see Note S1). Consequently, without consuming additional solar and droplet energies, or tailoring dielectric properties, the non-planar device consists of polyvinylidene difluoride (PVDF) and polytetrafluoroethylene (PTFE), enabling solar heat and raindrop energy harvesting with an output power increment of 174.3% and 65.4%, respectively. Together with scalable manufacturing techniques, we demonstrate pragmatic, weather-adaptive outdoor electricity generation on sunny, cloudy, night and rainy days. These results not only pave a new way for environmental heat/rain recovery but also for inspiration in other high-entropy energy utilization.

## RESULTS AND DISCUSSION

The conceptual mechanistic of a weather-adaptive energy harvester for solar heat and rain droplet recovery is schematically presented in Fig. 1. From a view of world climate regions, there are various seasonal kinds of weather at the equator, as well as the northern and southern hemispheres (Fig. S2) [39,40]. These distinct season-/climate-dependent weathers offer omnipresent sources for adaptive, complementary, and sustainable environmental energy harvesting in the form of solar heat, waste heat, wind energy and raindrop energy (Fig. 1a). However, unlike conventional solar heat and droplet-based energy harvesters, where planar dielectrics are employed with homogeneous heat dissipation and limited water spreading area/direction, in Fig. 1b we delivered a non-planar structure to confine the incident solar irradiance at a local area for increased transverse non-uniform heat propagation and variation (d*T*/d*t*), thus facilitating the dipole moment change towards pyroelectricity multiplication. Meanwhile, the curved bulk dielectric provides an enlarged lateral surface area for contact triboelectrification at droplet-PTFE interfaces, which, likewise, the non-uniform droplet spreading on the non-planar surface results in higher triboelectric output than typical flat architectures. Experimentally, the non-planar design boosts heat harvesting performance with an output power enhancement of 174.3% at 0.2 sun (Fig. 1c), which surpasses that of reported conventional planar pyroelectrics (for detailed calculations see Table S1) [27–30,41]. In addition, the developed non-planar dielectric increases rain droplet electricity generation by 65.4% under consistent input. This facile but effective weather-adaptive energy harvesting strategy, with the possibility for scalable manufacturing, holds enormous potential for advancing energy efficiency and autonomy in system operation.

### Non-planar dielectrics enhanced solar heat harvesting

**Figure 2.**
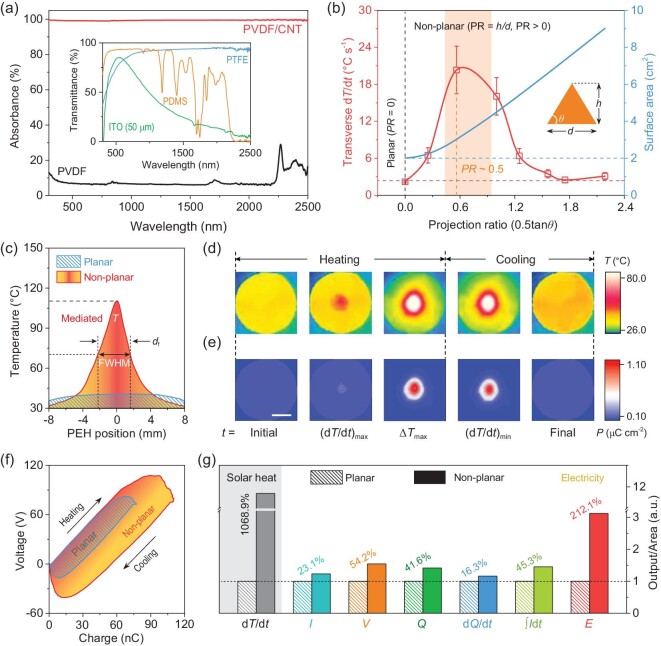
Non-planar dielectric enhanced solar heat harvesting. (a) Absorbance and transmittance of non-planar dielectrics. (b) Measured transverse d*T*/d*t* of PVDF/CNT films and surface area versus projection ratio of conical dielectrics. The surface area was estimated from the curved lateral surface area at different projection ratios. The dashed line indicates the transverse d*T*/d*t* and surface area of planar dielectrics (PR = 0). The solar irradiance is 0.1 sun. (c) Temperature distribution of planar and non-planar PEH units at 0.2 sun irradiation. The upper and lower dashed lines stand for the maximum and half maximum temperatures. FWHM refers to the full width at half maximum of the temperature profile. (d) Inhomogeneous spatiotemporal temperature and (e) polarization distribution evolution of a non-planar PEH unit at initial, maximum d*T*/d*t*, maximum Δ*T*, minimum d*T*/d*t*, and final stages. Scale bar: 5 mm. (f) The voltage varies with charge and harvested energy (presented in the coloured integral area) of a single planar and non-planar PEH unit. (g) The comparison of thermal and electrical outputs of planar and non-planar PEH units. The output of planar PEH is normalized at 1.0 (dashed line) for reference. The d*Q*/d*t* and ∫*I*d*t* stand for the time-differential of measured charge and time-integral of measured current, respectively.

**Figure 3.**
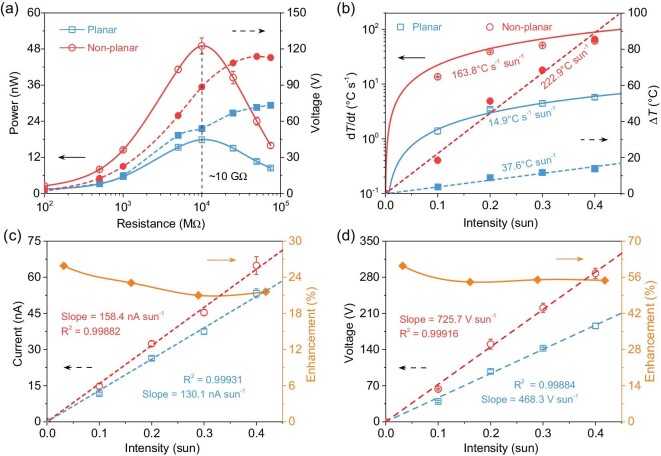
Load measurement and intensity dependence of non-planar solar heat harvesters. (a) Output power (left, solid lines) and voltage (right, dashed lines) of planar and non-planar PEH units at varied load resistances under 0.2 sun. The matched load resistance (∼10 GΩ) for two PEH units is consistent. (b) Temporal temperature changes (left, solid lines) and temperature gradients (right, dashed lines) of planar and non-planar dielectrics at different intensities. (c) Current (left, dashed lines) and corresponding output enhancement (right, solid line) of planar and non-planar PEH units at different intensities. (d) Voltage (left, dashed lines) and corresponding output enhancement (right, solid line) of planar and non-planar PEH units at different intensities.

To elucidate how the non-planar dielectric impacts solar heat harvesting and underlying pyroelectric characteristics, a poled-PVDF thin film coated with a carbon nanotube (CNT) solar absorber (Fig. 2a) was employed as the pyroelectric energy harvester (PEH) for photothermal measurements (Note S2). A conical bulk dielectric with transparent layered structures, i.e. PTFE, indium tin oxide (ITO), or polydimethylsiloxane (PDMS), was utilized to confine the incident solar heat onto the PEH surface (Fig. S3). The low transmittance of ITO in the wavelength of >750 nm is mainly ascribed to a large thickness of 50 μm, which can be further increased by using thinner films via advanced deposition techniques [42–44]. Thereby, the temporal temperature fluctuation (d*T*/d*t*) at the focused hotspot can be modulated by the projection ratio (PR, PR = height/diameter, half of the tangent value of bottom angle *θ*) of the conical dielectric with varied geometries. Notably, a maximum local d*T*/d*t* increment of 757.7% (PR ≈ 0.5, corresponding *θ* = 48°) is achieved owing to efficacious photothermal confinement and heat dissipation, compared with the planar device (PR ≈ 0) under 0.1 sun illumination (Fig. 2b, Fig. S4). Meanwhile, the enlarged PTFE lateral surface area of curved structures with a large PR value promotes unrestrained droplet spreading while rarely deteriorating sunlight transparency (Fig. 2a and b) [8,16,45]. Consequently, the non-planar dielectric with a PR value of 0.5 (i.e. height = 8 mm, diameter = 16 mm, corresponding *θ* = 45°) was employed to perform thermal and electrical measurements (Note S2). The measured temperature profile of the non-planar PEH reveals a temperature increase of 172.5% and transverse d*T*/d*t* by one order of magnitude (∼10 folds) higher at the irradiated local hotspot, in comparison to the uniform temperature profile of planar PEH (Fig. 2c, Figs S4b and S5b). Additionally, the temperature FWHM (full width at half maximum) of non-planar PEH is comparable to the focus diameter (*d*_f_ ≈ 4 mm), validating the phenomenon of solar-to-heat confinement and well-manipulated self-propagating thermal field across the device. In terms of spatiotemporal heat distribution of non-planar PEH under heating/cooling processes (Fig. 2d), the time-dependent multiple temperature contours at initial, (d*T*/d*t*)_max_, Δ*T*_max_, (d*T*/d*t*)_min_, and final stages affirm the delineated in-plane heat propagations from the hotspot to surrounding areas. The observed inhomogeneous heat transfer modulated by non-planar dielectrics differs from the typical thickness-dependent nearly constant heat dissipation under uniform solar heating. Furthermore, the pyroelectric coefficient (Fig. S5c) of a single PVDF-based film was measured to quantify the extent of polarization or surface charge density attainable from transverse heat variation, as demonstrated in Fig. 2e. The spatial polarization profiles at the 5 corresponding stages conform to the temporal transverse thermal field propagation (Fig. 2c and d) and present a non-uniform profile around one order of magnitude higher than the conventional polarization in the irradiated area (Fig. S6). Consequently, the overall enlarged heat variation and polarization of the non-planar PEH not only counteract the decrease in output in the non-illuminated area but also manifest the enhancement of the electrical output (Fig. 2f and g). As plotted voltage-charge integration (Fig. 2f), the work done, or energy harvested, can be estimated by the electrical potential (*V*) and transfer charges (*Q*) in a PEH capacitor [46]. Specifically, under fixed solar heating/cooling processes, the *V*−*Q* characteristics of non-planar PEH indicate considerably higher voltage (150.0 V) and charge accumulations (108.6 nC), as well as collected energy (6.8 μJ) than the planar device (Fig. S7). Also, the load measurement verified the advanced power generation of non-planar dielectrics for a single PEH unit with an enlargement of 174.3% (Fig. 3a, Note S3), offering a peak power density of 6.1 mW m^−2^ at 0.2 sun (Table S1). Then, we measured the thermal and electrical output of a non-planar PEH unit at different intensities to identify its heat harvesting performance for practical variable weather scenarios. Notably, the d*T*/d*t* of planar and non-planar devices changed linearly with respect to light intensities, and the corresponding enhancement is of 2 orders of magnitude (∼999.3%) compared with conventional planar designs. Meanwhile, the increment of linear intensity-dependent Δ*T* by employing a non-planar design is around 492.8%, suggesting highly efficient solar-to-heat conversion (Fig. 3b). These substantial increments in local thermal field manipulation are beneficial for current and voltage multiplications. Specifically, the intensity-dependent current output was boosted from 130.1 nA sun^−1^ (planar) to 158.4 nA sun^−1^ (non-planar), with an average enhancement of around 24% (Fig. 3c). Similarly, the intensity-dependent voltage output increased to over 50% solely by introducing non-planar dielectrics (Fig. 3d). These results agree well with the pyroelectric formulae, in which the voltage and current varied linearly with respect to the incident intensity, and the non-planar one showed a higher output, indicating more efficacious heat-to-electricity harvesting performance under consistent solar input without material property tailoring or energy penalty. Accordingly, the non-planar PEH demonstrates a comprehensive advancement both in thermal and electrical outputs far greater than the planar device.

### Non-planar dielectrics boosted droplet energy harvesting

**Figure 4.**
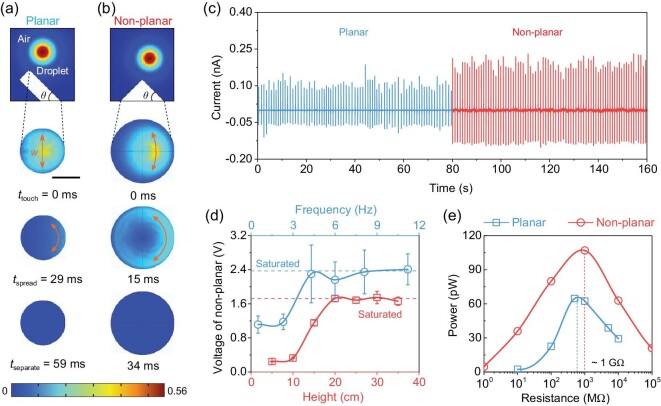
Non-planar dielectrics boosted raindrop energy harvesting. (a) Finite element modelling of droplet dynamics on planar and (b) non-planar PTFE films, where *t*_touch_, *t*_spread_, and *t*_separate_ stand for the droplet initial touching time, time at droplet maximum spreading area, and final separation from the PTFE surface, respectively. The colour refers to the water volume fraction. *θ* = 45°, scale bar: 10 mm. (c) Measured current output of planar and non-planar TEH units. (d) Measured voltage of non-planar TEH under various dropping heights and frequencies. (e) Load measurement of planar and non-planar TEH units at consistent dropping conditions.

We further characterized the surface textures of the proposed non-planar energy harvester, as shown in Fig. S8. The microscopic surface morphology/roughness of PTFE films enables the local triboelectric charge density to be enhanced [47–49], while scarcely deteriorating the overall sunlight transparency of the device (Fig. 2a). Also, the co-designed non-planar dielectrics with a tilting angle of 45° (Fig. 2b) facilitates the temporal droplet spreading area changes towards efficacious interfacial triboelectrification [8,34,36]. These advantages demonstrate a desirable water droplet energy collection potential. The mechanistic of rain droplet energy harvesting kinetics based upon single-electrode mode during spreading/separation [50,51] is schematised in Fig. S9. Initially, the falling water droplet impinges the hydrophobic PTFE dielectric and spreads unevenly to offer a non-uniform triboelectric charge density profile at liquid-solid non-planar interfaces. This charge density difference induces interfacial charge transfer from the PTFE film to the droplet (Fig. S9a and b), and the remaining charge of the PTFE film is re-balanced and conducted to the output terminal by ITO back electrodes (Fig. S9c and d). Thereby, the triboelectric signal is an instantaneous output governed by the droplet spreading and separation dynamics on the surface of solid dielectrics. Moreover, we performed finite element modelling using COMSOL Multiphysics software (Note S2 and Table S2) to elucidate the possible curvature-dependent water droplet spreading dynamics. From a view of surface wetting kinetics and electrostatic induction at droplet-PTFE liquid-solid interfaces, the electrical output of a triboelectric energy harvester (TEH) is governed by the temporal spreading area change (d*S*/d*t*) [36]. Here the d*S*/d*t* can be indirectly estimated from the wetting arc (*w*) and correlated perpendicular droplet spreading velocity [52,53]. The falling droplet typically spreads onto the dielectric surface upon touching and then separates within a few milliseconds. Distinct from conventional planar units, where the water droplet spreads on a limited flat PTFE surface along a fixed tilting angle/direction, thereby resulting in a small wetting arc and spreading factor (Fig. 4a, Fig. S10), by contrast, the non-planar dielectric with a curved surface facilities droplet spreading with a large wetting arc towards d*S*/d*t* increment of 109.7%. Consequently, the enlarged d*S*/d*t* benefits the efficient charge transfer between the water droplet and dielectrics under triboelectrification and electrostatic induction for increased electricity enhancement [36,54,55]. In the experimental verification, the droplet spreading process of planar and non-planar TEH units was also captured using a high-speed camera (Note S2 and Fig. S11). Notably, not only the spreading area (orange dashed) of the droplet at a non-planar dielectric curved surface is greater than that of a planar surface but also the total contact time (∼21 ms) is reduced significantly compared with the planar structure (∼35 ms). These findings are consistent with the simulation results (Fig. 4a and b) and manifest the priority of non-planar dielectrics for droplet-based energy harvesting though the correlation between contact time and non-planar dielectrics (e.g. droplet dynamics versus curvature, surface texture, and local triboelectric charge density) remains further investigations. Moreover, the output current (Fig. 4c) of non-planar TEH is around one-fold higher than that of the planar unit at consistent deionized water droplet volume (20 μl), dropping height (20 cm) and dropping frequency (1 Hz) (Note S2). However, in practical raindrop energy harvesting, the dropping height is infinite, and the dropping frequency is rainfall-dependent and uncontrollable. Therefore, we varied the dropping height and frequency separately to determine the maximum electrical output of non-planar TEH units. Interestingly, the voltage output tends to saturate and stabilize under increased dropping heights and frequencies (Fig. 4d, Figs S12 and S13). These results are mainly attributed to the electrostatic charge saturation under continuous droplet impinging and substantially simplify the implementation of *in-situ* raindrop energy harvesting [34,56–58]. Finally, the output power of a single non-planar and planar TEH unit was estimated (Fig. 4e, Note S3). The slight increase in internal impedance of the non-planar TEH could be ascribed to the decrease in average capacitance due to the introduction of thick PDMS conical/non-planar dielectrics [59,60]. The achieved 0.1 nW power output of the non-planar unit results in a 65.4% enhancement compared to the planar device, which can be further improved by scaling up the device with multiple TEH units using advanced manufacturing technologies.

### Scalable energy harvester and outdoor test

**Figure 5.**
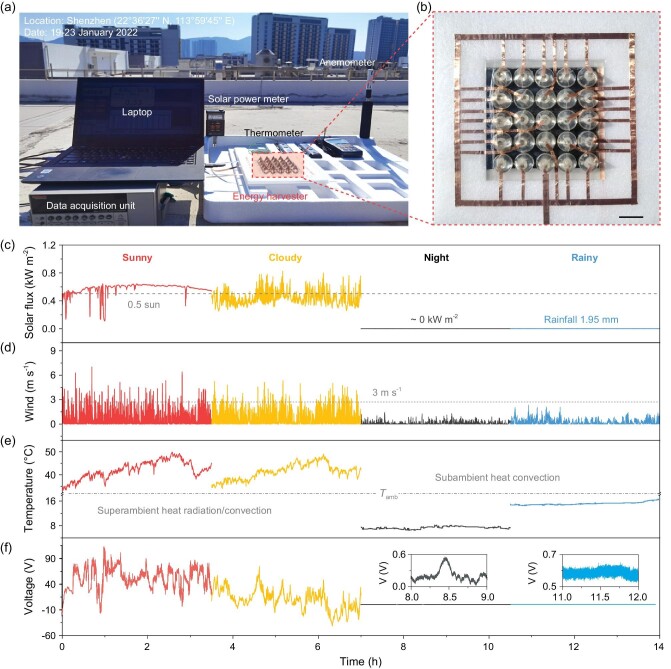
Outdoor test of scalable prototype for weather-adaptive energy harvesting. (a) Outdoor real-time test set-up. (b) Photograph of the scalable energy harvester. Scale bar: 2 cm. (c) Solar flux, (d) wind speed, (e) PVDF surface temperature, and (f) output voltage of scalable prototype under sunny, cloudy, night and rainy conditions.

The proposed all-in-one non-planar dielectric with weather-adaptive complementary energy harvesting capability also shows desirable scalability aided by additive manufacturing. The flowchart of a 3D-printed device is delivered in Fig. S14, where the triboelectric and pyroelectric dielectrics were deposited layer-by-layer followed by the optimized geometry diameters (Fig. 2b, PR = 0.5). Specifically, a piece of polymethylmethacrylate (PMMA, acrylic) with an optimum thickness of 16 mm (i.e. lens-PEH distance = 16 mm) covered with mask A was utilized for selective PDMS conical lens deposition (height = 8 mm, diameter = 16 mm). Then, multiple holes (diameter = 10 mm) with periodic array were obtained via laser cutting aided with mask B for focused sunlight transmission. After that, ITO electrodes and copper wires were deposited separately with masks B and C using a plasma coater, together with painted PTFE thin film to complete the fabrication of the triboelectric energy harvester. For the scalable printing of sandwiched pyroelectric devices, CNT electrode layers were formed via the evaporative coating method before and after a commercial poled-PVDF thin film (100 mm × 100 mm × 80 μm) on the back surface of the as-fabricated triboelectric module. To demonstrate the scalability, stability, and practicability of the proof-of-concept prototype for weather-adaptive environmental energy harvesting, we performed *in-situ* outdoor tests on sunny, cloudy, night and rainy days, respectively (Note S2). The solar flux, wind speed, output voltage, ambient temperature and relative humidity, were recorded concurrently (Fig. 5, Fig. S15). The PVDF thin film, incorporated with 25 conical TEH units (in parallel), was utilized for measurement. The ITO lines were replaced with copper conductive tapes to simplify the electrode line preparation (Fig. 5b), while the ITO conductive electrodes of non-planar devices were retained. The electrode lines were solely utilized to electrically connect the individual non-planar TEH, and the influence of copper electrodes (with a dimension of 2.0 mm × 11.3 mm × 50.0 μm, with a surface coverage area proportion of 11.9% for each unit) on photothermal collection and power generation is relatively minimal. The pyroelectric output induced by PVDF surface temperature fluctuations is driven by naturally confined solar illuminations and surrounding wind-/humidity-driven heat convection (Fig. 5c and f). Notably, the measured maximum instantaneous voltages are around 121.1 V (sunny) and 89.5 V (cloudy) at a mere 0.5 sun and wind speed of 3 m s^−1^ (Fig. 5c, d and f), respectively. Such common but overlooked superambient heat variations under day-to-day illumination most of time in non-equator countries/areas is feasible to sustain consumer electronics and sensors (power demands in a range of nW to μW) [6,21]. Subambient heat convection (without illumination) also generates a maximum voltage of ∼0.43 V at night using PVDF films. Moreover, on rainy days, the droplet electricity generation of a peak voltage of 0.1 V was attained at a rainfall of 1.95 mm and can be further boosted under heavy rainfall (Fig. 4d, Fig. S13). The electricity harvested was estimated to be 4.571 mJ in 14 hours, which is comparable to that of reported environmental energy harvesters [34,54]. These results offer a complementary capability for harvesting rain droplet energy using the all-in-one non-planar electricity generator. Though the output power from raindrop energy harvesting is low due to the intrinsic inefficiency of electrostatic induction under a single-electrode model [56] and slight rainfall, it is still greater than conventional planar designs and can be upgraded with advanced manufacturing techniques [34]. Moreover, we conducted simultaneous outdoor tests of PVDF-based devices and solar cells in cloudy and night conditions (details see Note S2 and Fig. S16). The voltage variation indicates that our PVDF-based pyroelectric device (with a peak voltage of ∼28.5 V) is more sensitive to converting surrounding heat fluctuations into electricity compared with the solar cell (1.05 V) both in windy and night scenarios. The PVDF-based pyroelectric device can harvest ambient convective heat variations even at night while there is no voltage output for solar cells. Consequently, our design could offer a feasible and complementary solution for solar heat utilization, enabling all-weather energy harvesting.

## CONCLUSION

We demonstrated well-manipulated thermal and electrostatic field inhomogeneity towards weather-adaptive solar heat and rain energy harvesting via an all-in-one non-planar electricity generator. The PTFE/ITO/PDMS-aided PVDF-based conical energy harvester achieves an output power increment of 174.3% and 65.4% for solar heat and raindrop energy scavenging, respectively, in comparison to conventional planar devices. This enhanced electrical output is attributed to transverse inhomogeneous heat propagation with enlarged pyroelectric polarization, as well as non-uniform droplet spreading-induced triboelectrification that overcomes the impediment and inefficiency of planar dielectrics. Further development in optically engineered curved dielectrics (e.g. lens array integration and size miniaturization), and liquid-solid triboelectric modes (freestanding triboelectric-layer, contact-separation), are promising to advance environmental energy harvesting. This weather-adaptive upcycling of low-grade heat and raindrop energy through controlled thermal and electrostatic field non-uniformity also provides insight into other new energy harvesting frontiers, e.g. passive photothermal manipulation, wave/wind energy modulation, transverse thermoelectrics, thin-film heat management.

## Supplementary Material

nwad186_Supplemental_File
