# Supplementary material for: Non-planar dielectrics derived thermal and electrostatic field inhomogeneity for boosted weather-adaptive energy harvesting
Source: Natl Sci Rev. 2023 Jun 28;10(9):nwad186. doi: 10.1093/nsr/nwad186 (PMC10411684; doi:10.1093/nsr/nwad186)
Supplement: nwad186_Supplemental_File [file nwad186_supplemental_file.pdf]

Supplementary data for

Non-planar dielectrics derived thermal and electrostatic field inhomogeneity for boosted weather-adaptive energy harvesting

Yi Zhou[1,2], Tianpeng Ding[2,3], Yin Cheng[2], Yi Huang[1], Wu Wang[1], Jianmin Yang[1,4], Lin Xie[1], Ghim Wei Ho[2,3,5,*], and Jiaqing He[1,6,*]

[1]Shenzhen Key Laboratory of Thermoelectric Materials and Department of Physics, Southern University of Science and Technology, Shenzhen 518055, China;

[2]Department of Electrical and Computer Engineering, National University of Singapore, Singapore 117581, Singapore;

[3]School of Electronic Science and Engineering, State Key Laboratory of Electronic Thin Film and Integrated Devices, University of Electronic Science and Technology of China, Chengdu 611731, China;

[4]Department of Materials Science and Engineering, National University of Singapore, Singapore 117575, Singapore;

[5]Institute of Materials Research and Engineering, A*STAR (Agency for Science, Technology and Research), Singapore 138634, Singapore;

[6]Guangdong-Hong Kong-Macao Joint Laboratory for Photonic-Thermal-Electrical Energy Materials and Devices, Southern University of Science and Technology, Shenzhen 518055, China

***Corresponding authors.** E-mails: elehgw@nus.edu.sg; hejq@sustech.edu.cn

Table of Contents

Note S1. Discussion on non-planar dielectrics for weather-adaptive energy harvesting 4

Note S2. Experimental section 6

Note S3. Load measurement of PEH and TEH units 10

Figure S1 Diagram of pyroelectric mechanism 11

Figure S2 Monthly varied rainy and non-rainy (sunny, cloudy, windy) days in Singapore, Shenzhen, Washington, and Cardiff across 12 months representing 4 climate zones 12

Figure S3 Solar heat redistribution and non-planar PEH measurement 13

Figure S4 Geometry-dependent solar heat modulation via non-planar conical lenses 14

Figure S5 (a) Optical image of Solar heat harvester. Scale bar, 0.5 cm. (b) PEH temperatures under heating/cooling conditions at 0.2 sun. (c) Pyroelectric coefficient of PVDF-based PEH unit 15

Figure S6 Measured temperature (a) and (b) polarization distributions of planar PEH unit at 5 stages under 0.2 sun illumination 17

Figure S7 Electrical output of planar and non-planar PEH units under periodic heating/cooling process at 0.2 sun 17

Figure S8 (a) Surface morphology, (b) roughness, and (c) wettability of PTFE films. 18

Figure S9 Schematic of droplet-based liquid-solid triboelectrification and electricity generation via axisymmetric conical dielectrics. 19

Figure S10 Finite element modelling of water droplet spreading kinetics on the surface of planar and non-planar PTFE films. 20

Figure S11 High-speed images of droplet spreading process at (a) planar and (b) non-planar dielectric surfaces 21

Figure S12 Voltage output of non-planar PEH unit at different dropping heights 22

Figure S13 Frequency-dependent voltage output measurement of non-planar TEH unit 23

Figure S14 Flow chart of 3D-printed scalable non-planar energy harvester. 24
Figure S15 Outdoor test of scalable prototype for weather-adaptive energy harvesting at Shenzhen, China 25
Figure S16 Outdoor simultaneous test of PV and pyro devices under cloudy and night conditions in Singapore 26
Table S1. Comparison of normalized power output for solar pyroelectrics 27
Table S2. Parameters for finite element modelling of planar and non-planar TEH units 28
Supplementary References...29

Note S1. Discussion on non-planar dielectrics for weather-adaptive energy harvesting

From the point of view of the Second Law of Thermodynamics, the total entropy of an isolated system can never decrease over time, so the disordered, decentralized, and distributed energy forms are widespread around the surroundings (Fig. 1a). In this context, the temporal temperature change (dT/dt), a ubiquitous thermal energy resource of greater abundance which arises from any non-static illuminations due to wind, cloud cover etc., is a cornucopia of energy sources with immense potential in the field of green energy to meet the climate target and sustainable development goals (SDG) [1-3]. In specific, there is commonly weather/season-dependent low-light irradiance, and rainfall fluctuates with wind speed and humidity changes [4-6]. Unfortunately, these non-static, weather-dependent, low-grade heat variations are of equal significance as the static temperature gradient while are far-flung and agelong negligence. More importantly, in terms of energy sustainability and carbon neutrality, the challenges are "how can we harness this energy that is all around us, but not yet utilized for much of anything" [7,8]. For instance, how to engineer the configuration and develop sustainable approaches from the material, device, and system levels, as well as nanoscale to macroscale views, to capture environmental underexplored energies into cost-effective electricity via an adaptive manner? Traditionally, the planar film configuration was utilized for environmental energy harvesting owing to bottom-up layer-by-layer structure design and facile fabrication procedure. However, the planar structure with a low degree of freedom is not a priority to meet high-entropy energy harvesting of diffused, disordered, decentralized, and distributed energy sources due to its high demand for structure adaptiveness and device efficacy. Explicitly, conventional planar pyroelectrics capture the solar heat homogenously, limited by simultaneous and uniform thermal field propagation across the entire device (Fig. S1) [6,9,10]. These approaches with spatiotemporally coupled thermodynamic processes restrain the heat variation and temperature

gradient (ΔT), thus resulting in limited pyroelectric output [11,12]. Meanwhile, traditional rain droplet-based electricity generators mainly utilize flat triboelectric dielectrics with a tilting angle via electrostatic induction and triboelectrification during the temporal liquid-solid contact/separation process along the sliding direction [13-18]. Thereby, one of the facing challenges in the domain of environmental energy harvesting is how to enhance the temporal heat variation and droplet spreading change adaptively via a facile configuration. According to pyroelectric fundamentals, an intense dT/dt from large ΔT contributes to creating a larger dipole moment shift (P_S), thus leading to higher electrostatic intensity induced surface charge density [11]. Also, the contact electrification mechanism suggests that the triboelectric output can be enhanced by promoting the temporal droplet spreading area change (dS/dt) [16]. Therefore, it is urged to develop non-planar dielectrics with a high degree of freedom for flexible manipulation of thermal and electric field propagation, aiming to boost the power output. Herein, we first focused on how the facile, in-plane, macroscopic heat modulation contributes to upcycle and rationalizes the inexhaustible but unusable, low-light solar irradiance into in-plane heat propagation towards enhanced P_S change and pyroelectric output without tailoring materials properties [19,20], altering pyroelectric coefficients [21], or applying electric fields [22]. We solely redistributed the incident light onto the PVDF-based film via a non-planar dielectric to trigger inhomogeneous heat propagation from the hotspot to non-irradiation areas along the transverse direction, thus achieving intense dT/dt and P_S changes (Figs 1b and 2d and 2e). Second, we analyzed how the non-planar dielectric promote droplet spreading area change on the enlarged, curved surface for high triboelectric output from experiments and simulations. Our findings verified the non-planar dielectric is capable of increasing the output by 1-2 folds for pyroelectricity and droplet-based triboelectricity (Fig. 1c).

Note S2. Experimental section

1.1 Device fabrication and characterization

A ferroelectric poly(vinylidene difluoride) (PVDF) (Fils Co., Ltd.) thin film with a dimension of 16 mm (diameter) × 80 μm (thickness) is utilized for solar heat harvesting. The top and bottom sides of the PVDF thin film were deposited with carbon nanotubes (CNT, Xianfeng Nano) electrodes using evaporation coating methods, and details can be found in the previous work [6,23]. These CNT solar absorbers also offer desirable solar-to-heat conversion efficiency. Then, two copper tapes (3.0 mm in width, 66 μm in thickness) were affixed to the top and bottom of PVDF for electrical output conductive wires. After that, the single pyroelectric unit was mounted onto a circular-matched hollow acrylic cantilever. Besides, a polymerized polydimethylsiloxane (PDMS, SYLGARD 184) conical lens was covered with indium tin oxide (50 μm in thickness, Langu Electronics Tech Co., Ltd.) and polytetrafluoretyhylene (50 μm in thickness, FGA-03, NITTO) thin films layer-by-layer on the side surface. The copper electrically conductive wire was attached to the ITO layer to form the single-electrode mode triboelectric device fabrication. Moreover, the solar absorbance and transmittance spectra of PVDF, PVDF/CNT, PTFE, ITO, and PDMS conical structures were obtained using a UV-VIS-NIR spectrophotometer (UV-3600, Shimadzu). The morphology, roughness, and wettability of the PTFE thin films were characterized using an SEM (Nova NanoSem450, FEI), AFM (MFP-3D Stand Alone, Oxford), and contact angle instrument (SDC-100, SINDIN), separately.

1.2 Pyroelectric and triboelectric measurements

The electrical measurement of the pyroelectric device was conducted in a semi-closed moisture-proof cabinet (controlled temperature and relative humidity), where a Xenon lamp (CEL-PE300E-3A, CEAULIGHT), an electrometer (6514, Keithley), as well as an IR camera (E50, FLIR Systems), were positioned inside. The Xenon lamp was employed as a solar simulator

with an irradiance intensity of ~0 to 1250 W m^{-2} under room temperature (~25 °C) and relativity humidity (~60%). The solar irradiance intensity was measured by a solar power meter (1333R, TES). An electronic shutter (GCI-73, Daheng Optics) and a PTFE/ITO/PDMS conical lens were introduced into the light trace for tuning the light on/off (heating/cooling) time duration and focused illumination intensity. The heating/cooling time duration ratio was fixed at 0.5 for the whole test (light on, 30s; light off, 60s). The back image distance (lens-PEH distance) is fixed at 16 mm for solar heat confinement. The projection area of the conical lens and PEH under light trace remains consistent. The 6514 electrometer, coupled with LabVIEW software, was utilized to record the electrical output continuously over time. Besides, the FLIR camera below the PEH, together with FLIR Tools+ software was used to capture the infrared image and surface temperature. The temperature value analyzed in this work is the average value processed from the irradiated area of the PEH unit. The error bar was calculated from the standard deviation of peak-to-peak outputs of 10 cycles under periodic heating/cooling for each measurement. The pyroelectric coefficient measurement was performed using the Byer-Roundy method under varied heating temperatures [24,25]. For triboelectric measurement, a single PTFE/ITO/PDMS conical unit (PR = 0.5, $\theta \approx 45°$) horizontally supported by a plastic tube was utilized for droplet energy harvesting. By contrast, a planar PTFE/ITO/PDMS unit was fixed at a plastic tube with a tilting angle of 45°. A syringe pump (XMSP-2C, Ximai Nanotech) and an acrylic tube (outer diameter 3 mm, inner diameter 2 mm) were used to control and generate water droplets. The dropping height of deionized water droplet (20 μl) was fixed at 20 cm for current and voltage measurement. The dropping frequency of 1 Hz was used for open-circuit and load measurement. The droplet spreading process at planar and non-planar TEH surfaces was captured using a high-speed camera (MEMRECAM HX-7s, NAC) at a typical recording speed of 5,000 frames per second. All the triboelectric measurement was performed in a Faraday case to minimize the electronic noise.

1.3 Finite element analysis of droplet spreading/separation

The droplet spreading/separation dynamics on the surface of the TEH unit were performed by the finite element analysis method using COMSOL Multiphysics coupled with Laminar Flow and Level Set modules. The measured TEH and droplet dimensions, as well as other parameters selected from the COMSOL materials library, were utilized in the simulation (Table S2).

1.4 Outdoor test of scalable weather-adaptive energy harvester

The outdoor test was conducted at the location of 22°36'27" N, 113°59'45" E, the roof of Block 10, Innovation Park, Southern University of Science and Technology campus, within a time duration of 10.30 AM to 2.00 PM, 19 January 2022, for sunny test; 1.00 AM to 4.30 AM, 20 January 2022, for night test; 10.30 AM to 2.00 PM, 21 January 2022, for cloudy test; 04.30 AM to 08.00 AM, 23 January 2022, for rainy test. The scalable all-in-one energy harvesters (25 TEH units, in parallel; one pyroelectric PVDF/CNT thin film) were arranged on a foam plate. For sunny/cloudy/night measurements, the sunlight irradiation together with wind/humidity-driven heat convection facilitates the hotspot temperature variation on the PVDF/CNT surface. In rainy conditions, the falling rain droplet impinges the PTFE conical array to induce triboelectricity. The electrical signal of pyroelectric and triboelectric devices was recorded using a 6514 electrometer. The PVDF surface temperature was monitored at the local hotspot by a temperature datalogger (SSN-61, YUWESE). The in-situ solar irradiation was measured using a solar power meter datalogger (1333R, TES). The ambient temperature, ambient relative humidity (RH), and wind speed were recorded by a hot-wire anemometer (1341, TES). The simultaneous outdoor test of a PVDF-based device and solar cell (polycrystalline silicon, TELESKY) with an identical area of 30 mm × 30 mm, was performed at the location of 1°17'56" N, 103°46'19" E, the grass ground behind E3-03-01, Multidisciplinary Lab, Kent Ridge campus, National University of Singapore, in the time duration from 08.00 PM (31 December 2022) to

8.00 PM (1 January 2023). In specific, the solar flux, voltage output of solar cell, ambient temperature, and PVDF temperature were recorded concurrently by using a four-channel datalogger (HD35EDLW wireless data loggers, DeltaOHM), and the generated voltage of pyroelectric PVDF-based device was acquired by using an electrometer (6514, Keithley) incorporated with LabView software. All data were collected simultaneously and connected to a laptop for processing.

Note S3. Load measurement of PEH and TEH units

The load measurement of planar and non-planar PEH units was conducted at a solar intensity of 0.2 sun with a heating/cooling (light on/off) time duration ratio of 0.5. A tunable load resistor (ZX79, Fuyang Precision) was serially connected with a PEH unit was used to vary the matched resistance and load voltage (load resistance was switched from 0.1, 0.5, 1, 5, 10, 25, 50, to 75 GΩ). The corresponding voltage signal was recorded using a 6514 electrometer under ten periodic heating/cooling cycles for each load measurement. Based upon Kirchhoff's voltage law and Ohm's law, the power (P_e) was extracted by time integration of voltage under corresponding load impedance (R) [26]

$$P_e = \frac{1}{t}\int_{t_0}^{t_0+t} \frac{V(t)^2}{R} \mathrm{d}t \tag{S1}$$

where the initial time is t_0, the integration time t was selected from the recorded time-dependent voltage signals. The calculated results using equation S1 are the average power in the time duration t, and not the peak power. Similarly, for load measurement of the planar TEH unit, the load resistance was switched from 0.01, 0.1, 0.5, 1, 5, to 10 GΩ. Moreover, for load measurement of the non-planar TEH unit, the load resistance was switched at 0.001, 0.01, 0.1, 1, 10, and 100 (unit: GΩ). The power density of TEH units is then calculated from formula S1. Apart from the average power density P_e, the peak power density (P_{max}) is also an evaluation matrix for directly determining the electrical output of energy harvesters. Typically, the P_{max} is dependent on the maximum current, voltage profiles, internal resistance, and load impedance. Specifically, the P_{max} is achieved as long as the internal resistance of the device equals the matched load resistance [26], as given by

$$P_{max} = \frac{I_{max} \cdot V_{max}}{4} \tag{S2}$$

where I_{max} and V_{max} represent the peak-to-peak current and voltage calculated from time-dependent signals at a specific time, respectively.

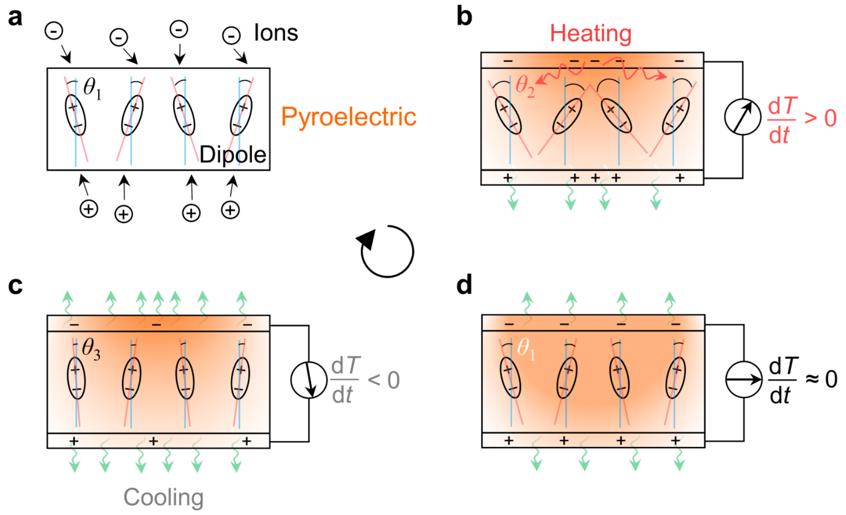

Figure S1 Diagram of pyroelectric mechanism. (a) Charges induced by the internal electric dipoles are neutralized by the ambient ions. **(b)** At the temporal heating state or dT/dt > 0, the electrostatic charges in the electrode induced by the internal dipole moment shift contribute to electrons flowing across the external circuit. **(c)** At a quasi-static thermo-equilibrium state or dT/dt = 0, the charges from the electric dipole moment are balanced by the electrodes, and no electrons flow. **(d)** At the temporal cooling state or dT/dt < 0, the reversely induced charges in the electrode by the internal dipole moment shift contribute to electrons flowing across the external circuit.

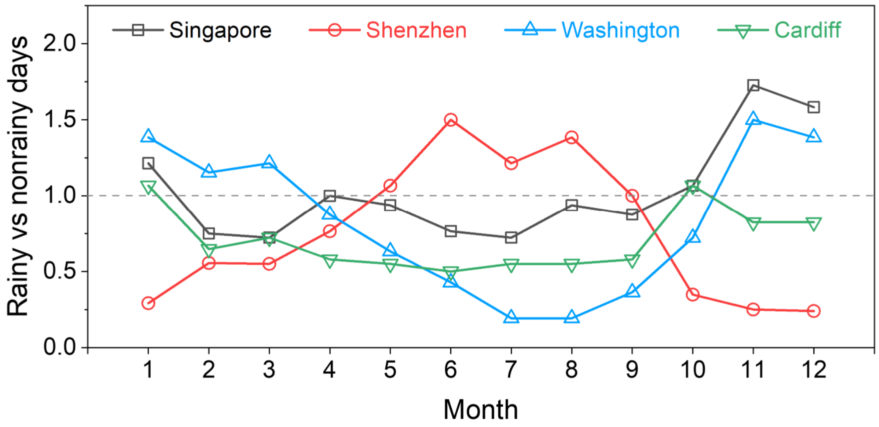

Figure S2 Monthly varied rainy and non-rainy (sunny, cloudy, windy) days in Singapore, Shenzhen, Washington, and Cardiff across 12 months representing 4 climate zones [27]

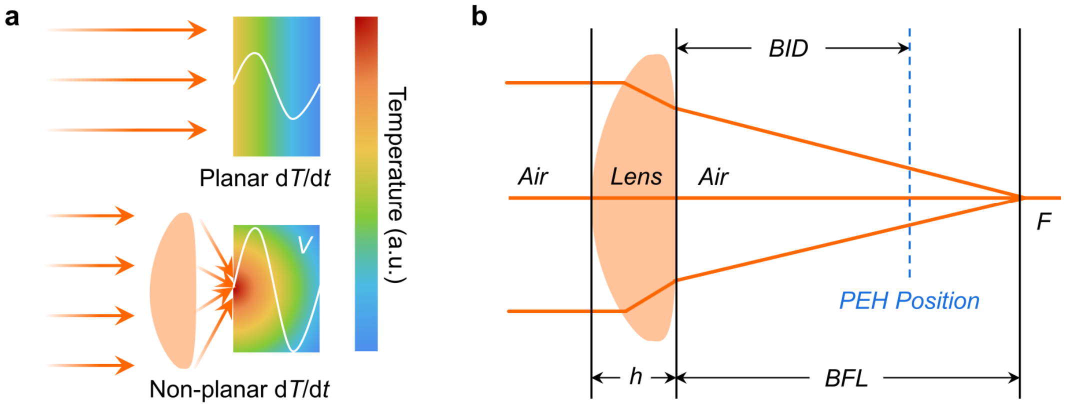

Figure S3 Solar heat redistribution and non-planar PEH measurement. (**a**) Schematic of solar heat conversion using planar and non-planar PEH units. (**b**) Geometric diagram of sunlight manipulation using a non-planar conical lens. BID: back image distance, i.e., PEH-lens distance; BFL: back focal length.

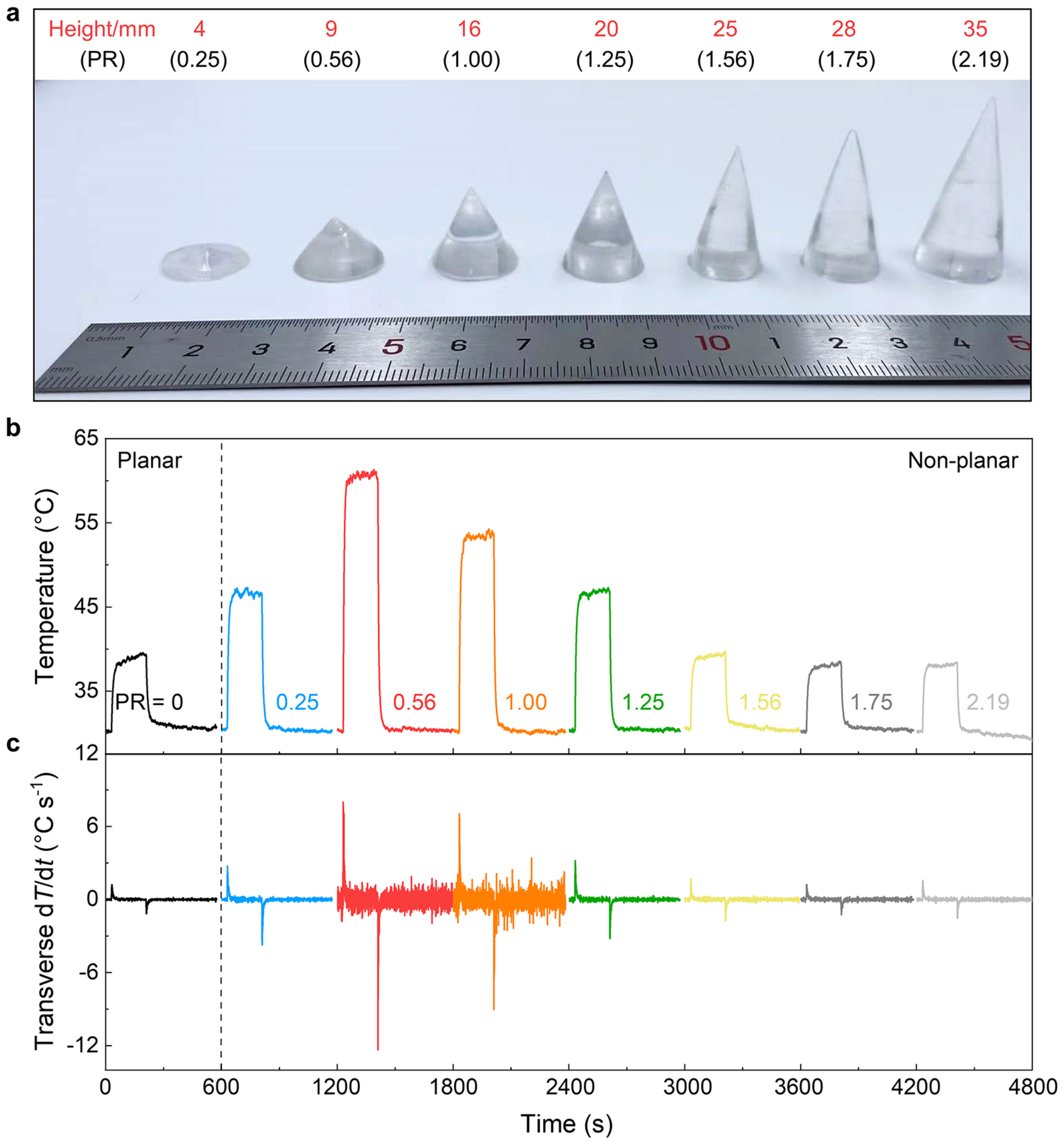

Figure S4 Geometry-dependent solar heat modulation via non-planar conical lenses. (**a**) Optical images of conical lenses with different heights. The diameter is 16 mm. PR = height/diameter. (**b**) Temperature and (**c**) temporal temperature variations at various projection ratios under 0.1 sun.

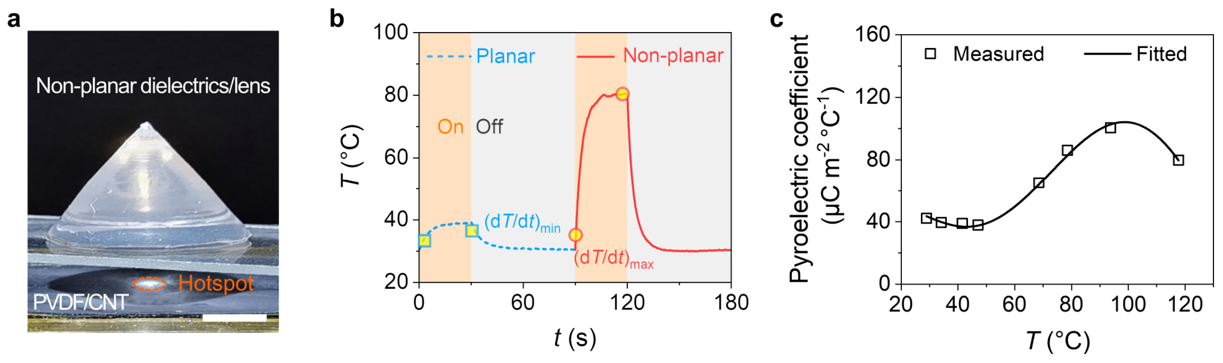

Figure S5 (a) Optical image of Solar heat harvester. Scale bar, 5 mm. (b) PEH temperatures under heating/cooling conditions at 0.2 sun. (c) Pyroelectric coefficient of PVDF-based PEH unit.

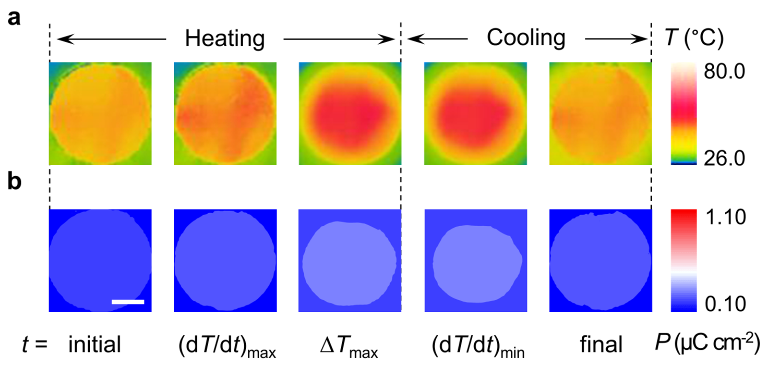

Figure S6 Measured temperature (a) and (b) polarization distributions of planar PEH unit at 5 stages under 0.2 sun illumination. Scale bar: 5 mm.

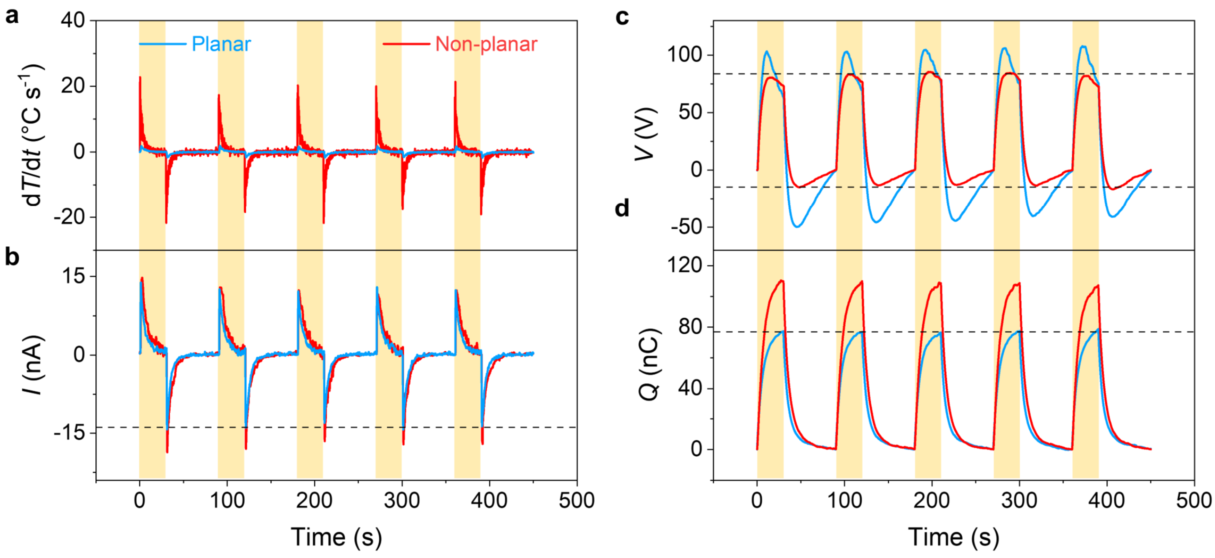

e

PEH unit	d*T*/d*t* (°C s^{-1})	*I* (nA)	*V* (V)	*Q* (nC)	d*Q*/d*t* (nA)	∫*I*d*t* (nC)	*E* (μJ)
Planar	3.5±0.2	26.4±0.8	97.6±1.6	76.7±1.0	29.3±0.8	75.8±2.6	2.2±0.2
Non-planar	40.5±6.5	32.4±3.5	150.5±3.2	108.6±2.3	34.1±2.1	110.1±5.8	6.8±0.7
Enhancement	1068.9%	23.1%	54.2%	41.6%	16.3%	45.3%	212.1%

Figure S7 Electrical output of planar and non-planar PEH units under periodic heating/cooling process at 0.2 sun. (**a**) Temperature, (**b**) current, (**c**) voltage, and (**d**) charge variations. (**e**) Comparison of thermal and electrical output for planar and non-planar PEH units.

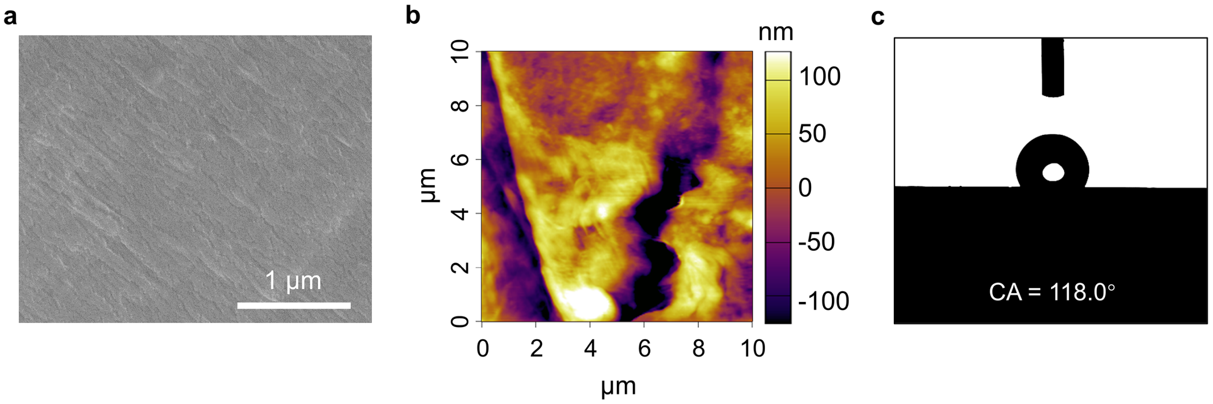

Figure S8 (a) Surface morphology, (b) roughness, and (c) wettability of PTFE films.

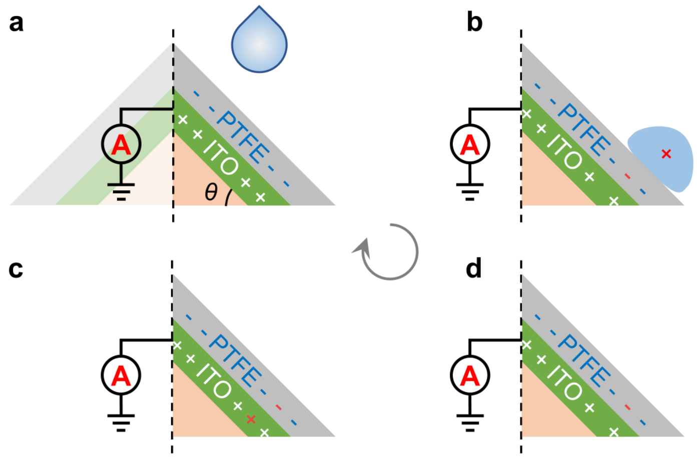

Figure S9 Schematic of droplet-based liquid-solid triboelectrification and electricity generation via axisymmetric conical dielectrics. The conical structure can be attained via axisymmetric rotation (dashed line) of the triangle plane.

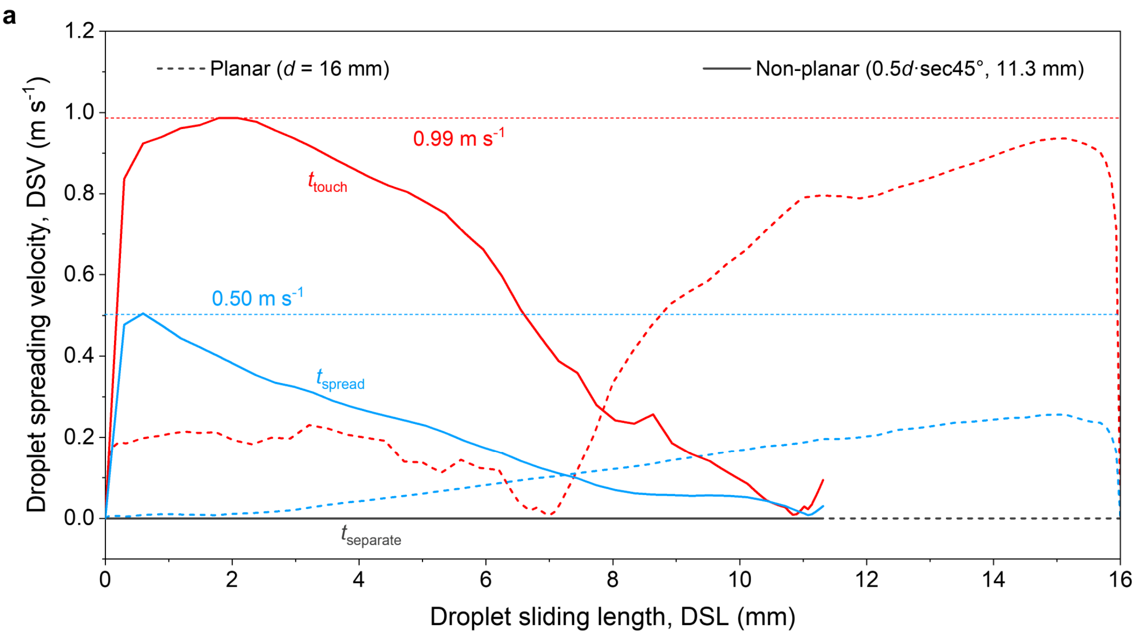

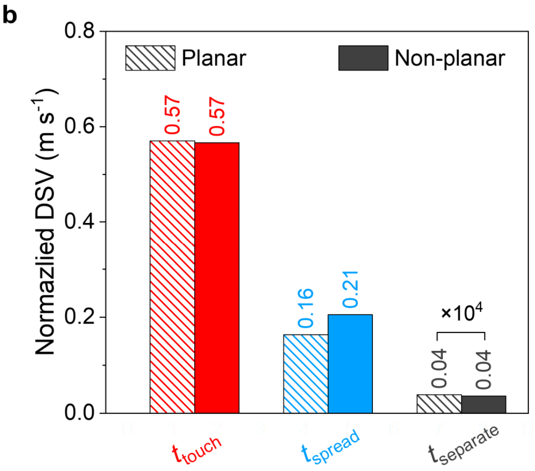

c

	Planar	Non-planar	Enhancement
Droplet	Volume 20 µl, diameter 3.37 mm		
PR	0	0.5	/
DSL_{spread} (mm)	16	11.3	29.4%
w (mm)	11.4	19.0	66.7%
DSV_{spread} (mm s^{-1})	163.5	205.0	25.4%
β	3.4	5.6	66.2%
dS/dt (cm^2 s^{-1})	18.6	39.0	109.7%

Figure S10 Finite element modelling of water droplet spreading kinetics on the surface of planar and non-planar PTFE films. (**a**) Droplet spreading velocity (DSV) versus droplet spreading length at different time. (**b**) Normalized DSV at touch, spread, separate conditions using planar and non-planar TEH units. (**c**) Comparison and enhancement of water droplet spreading and spreading area variations (dS/dt), where the spreading dS/dt is calculated from the wetting arc (w) and DSV_{spread}, the spreading factor (β) is defined as the ratio between w and water droplet diameter at initial state [16,28,29].

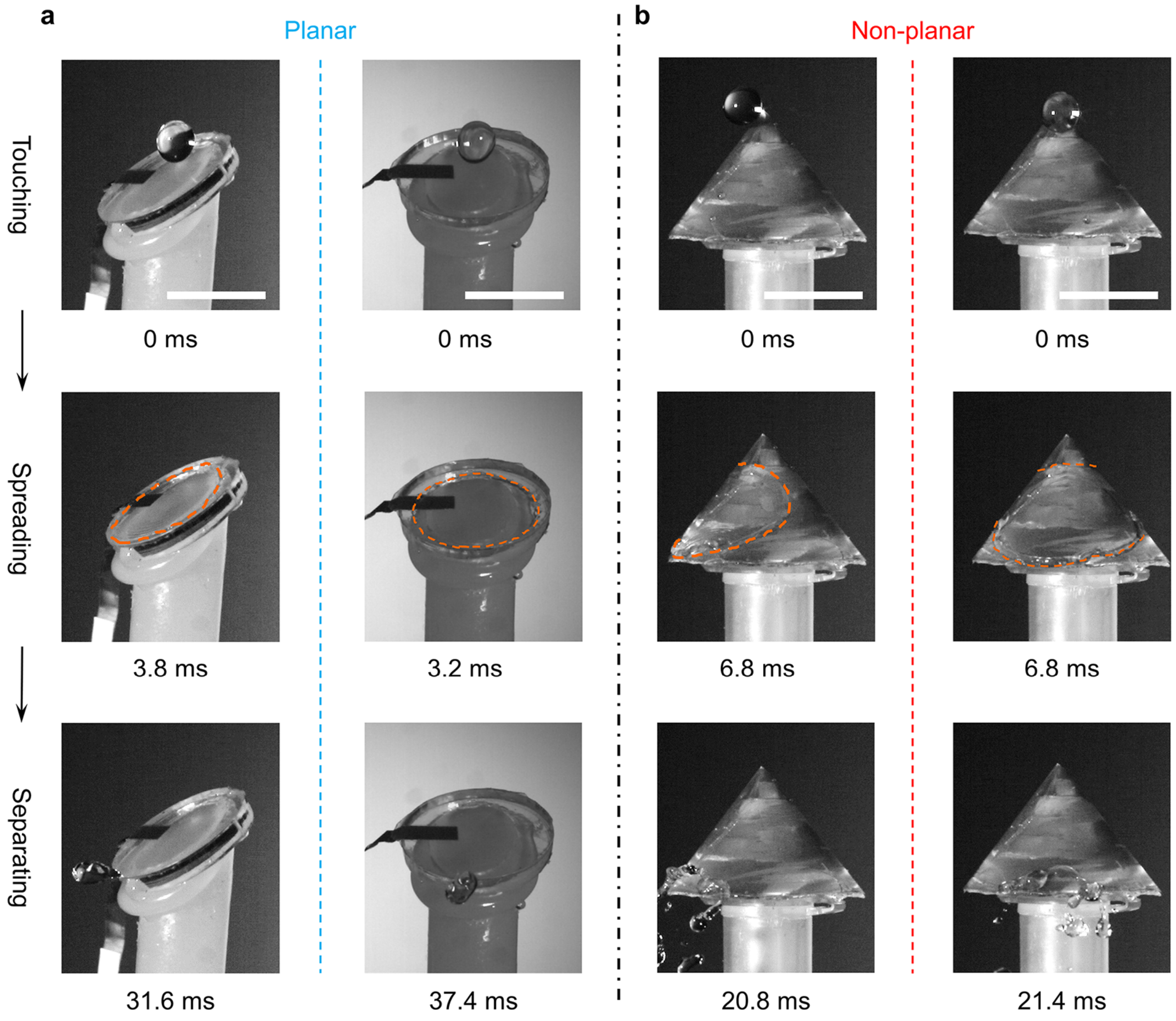

Figure S11 High-speed images of droplet spreading process at (a) planar and (b) non-planar dielectric surfaces. The side and front views are captured separately. Scale bar, 10 mm. Orange dashed lines refer to the boundary of maximum droplet spreading area. The recording speed is 5,000 frames per second.

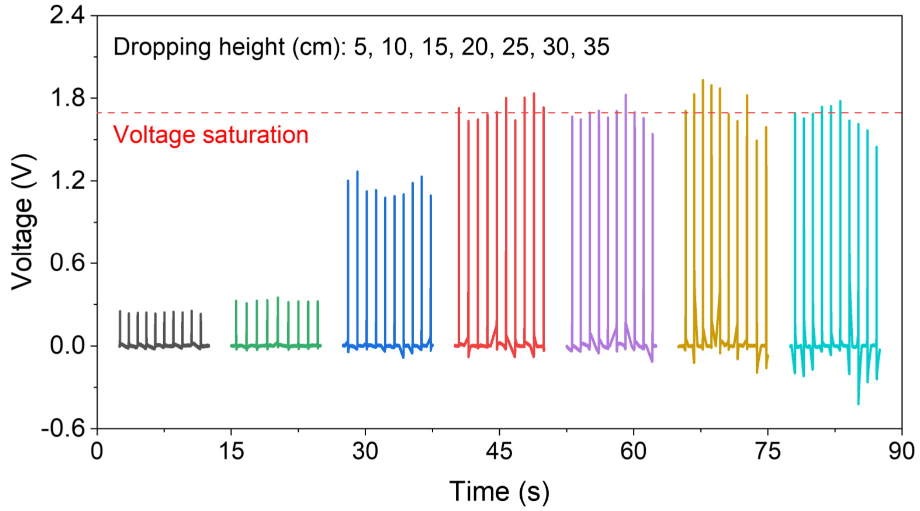

Figure S12 Voltage output of non-planar PEH unit at different dropping heights. The dropping frequency is 1 Hz.

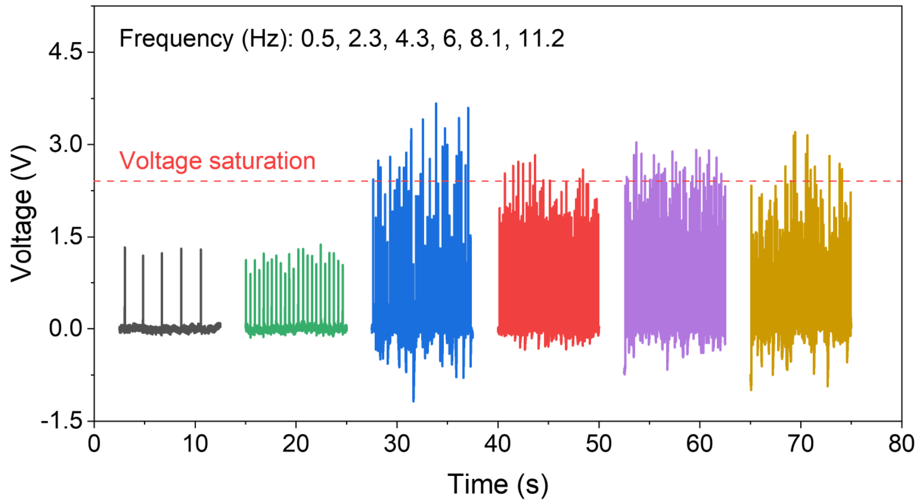

Figure S13 Frequency-dependent voltage output measurement of non-planar TEH unit. The dropping height is 20 cm.

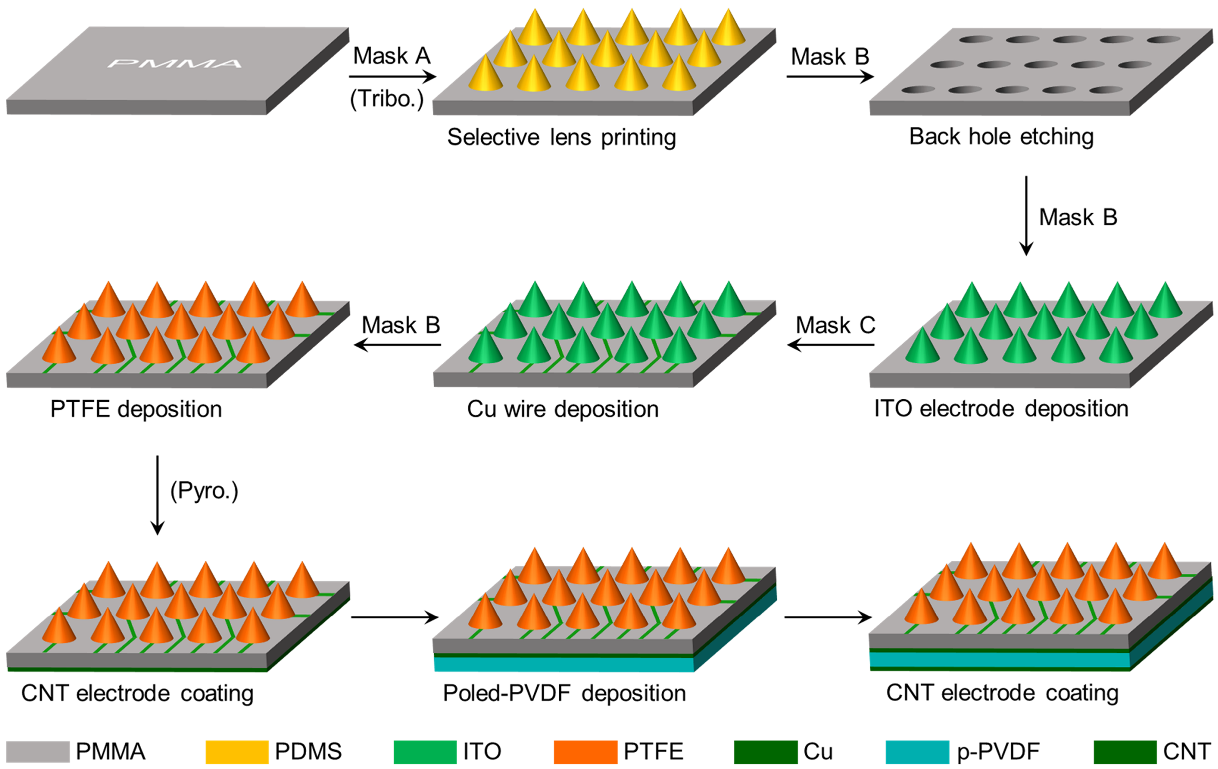

Figure S14 Flow chart of 3D-printed scalable non-planar energy harvesters.

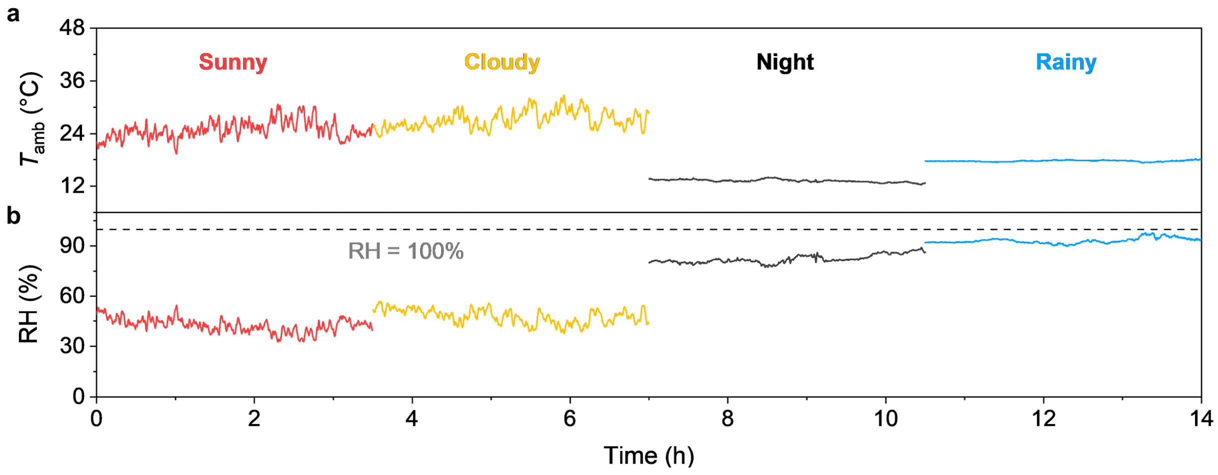

Figure S15 Outdoor test of scalable prototype for weather-adaptive energy harvesting at Shenzhen, China. (**a**) The ambient temperature and (**b**) RH for the outdoor test of scalable non-planar energy harvester system.

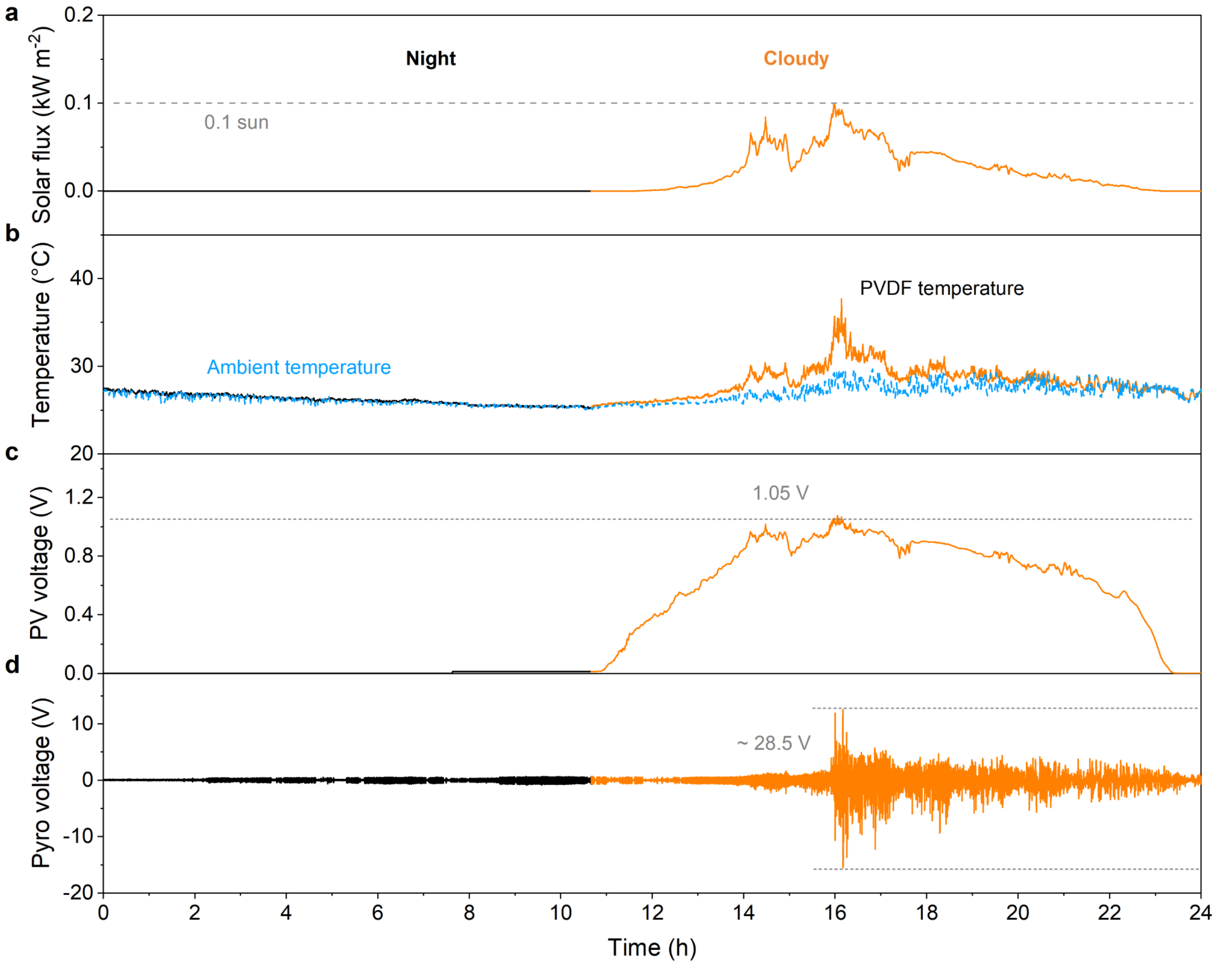

Figure S16 Outdoor simultaneous test of PV cell and PEH devices under cloudy and night conditions in Singapore. (**a**) Solar flux, (**b**) temperature, (**c**) PV voltage, and (**d**) pyro voltage.

Table S1. Comparison of normalized power output for solar pyroelectrics

PEH unit composition	Dimension a×b×c (mm^3)	Solar intensity ($kW\ m^{-2}$)	Peak Power density ($W\ m^{-2}$)	Planar/ Non-planar	Output/ Area	References
PVDF	75×75×0.08	1.00	180.0μ	Planar	0.36	[30]
PVDF	20×20×0.11	3.06	22.0μ	Planar	1.45E-2	[31]
PVDF	20×15×0.05	7.78	174.0μ	Planar	4.49E-2	[32]
PVDF	20×5×0.10	14.5	27.0μ	Planar	3.74E-3	[33]
BTO	Φ20×0.85†	0.25	48.0μ	Planar	0.38	[34]
PVDF	Φ16×0.08†	0.2	88.9μ (3.2m‡)	Planar	0.89	***This work***
PVDF	Φ16×0.08†	0.2	244.0μ (6.1m‡)	Non-planar	2.45	***This work***

† where ϕ stands for the diameter of circular PEH units, the power density was estimated from the formula S1, and the output/area was calculated from the average power density at 1 sun illumination. ‡ The power density was estimated from formula S2 and Fig. S7e. The apex angle of the non-planar sample is around 90° (PR = 0.5, corresponding θ = 45°), and the wettability (contact angle) of the PTFE surface is around 118° (Fig. S8c). The dropping height of deionized water droplet (20 μl) was fixed at 20 cm and the dropping frequency was 1 Hz (Note S2). The Weber number (We) of impacting droplets in the experimental conditions was estimated to be 180.8 from $\rho v^2 L/\gamma$, where ρ is the density, L is the characteristic length or droplet diameter, v is the impact velocity, and γ is the surface tension of droplets [14].

Table S2. Parameters for finite element modelling of planar and non-planar TEH units

Parameters	Planar	Non-planar
Droplet volume (μl)	20.0	
Dropping height (cm)	20.0	
Diameter of PTFE/ITO/PDMS (mm)	16.0	
Contact angle (°)	118.0	
Projection ratio/PR	0	0.5
Ambient temperature/T_{amb} (°C)	25.0	
Ambient relative humidity/RH (%)	60	

Supplementary References

1. International Energy Agency. *Energy Technology Perspectives 2020.* https://www.iea.org/reports/energy-technology-perspectives-2020 (1 March 2023, date last accessed)
2. Editors. A truly sustainable future. *Nat Sustain* 2022; **5**: 281.
3. Yang Y. Pyroelectricity gain in multilayers. *Nat Energy* 2022; **7**: 1007-8.
4. Johnson I, Choate WT and Davidson A. *Waste Heat Recovery. Technology and Opportunities in U.S. Industry*. Laurel, MD: BCS, Inc., 2008.
5. Peng Y, Fan L and Jin W *et al.* Coloured low-emissivity films for building envelopes for year-round energy savings. *Nat Sustain* 2022; **5**: 339-47.
6. Ding T, Zhu L and Wang X-Q *et al.* Hybrid photothermal pyroelectric and thermogalvanic generator for multisituation low grade heat harvesting. *Adv Energ Mater* 2018; **8**: 1802397.
7. Wang ZL. Entropy theory of distributed energy for internet of things. *Nano Energy* 2019; **58**: 669-72.
8. Yang Y and Wang ZL. Emerging nanogenerators: powering the internet of things by high entropy energy. *iScience* 2021; **24**: 102358.
9. Hanrahan B, Easa J and Payne A *et al.* A Portable power concept based on combustion and pyroelectric energy conversion. *Cell Rep Phys Sci* 2020; **1**: 100075.
10. Gao F, Li W and Wang X *et al.* A self-sustaining pyroelectric nanogenerator driven by water vapor. *Nano Energy* 2016; **22**: 19-26.
11. Bowen CR, Taylor J and LeBoulbar E *et al.* Pyroelectric materials and devices for energy harvesting applications. *Energ Environ Sci* 2014; **7**: 3836-56.
12. Korkmaz S and Kariper İA. Pyroelectric nanogenerators (PyNGs) in converting thermal energy into electrical energy: fundamentals and current status. *Nano Energy* 2021; **84**: 105888.
13. Lin ZH, Cheng G and Lee S *et al.* Harvesting water drop energy by a sequential contact-

electrification and electrostatic-induction process. *Adv Mater* 2014; **26**: 4690-6.

14. Xu W, Zheng H and Liu Y *et al.* A droplet-based electricity generator with high instantaneous power density. *Nature* 2020; **578**: 392-6.
15. Zhang J, Lin S and Zheng M*, et al.* Triboelectric nanogenerator as a probe for measuring the charge transfer between liquid and solid surfaces. *ACS Nano* 2021; **15**: 14830-7.
16. Wang X, Fang S and Tan J *et al.* Dynamics for droplet-based electricity generators. *Nano Energy* 2021; **80**: 105558.
17. Gang X, Guo ZH and Cong Z *et al.* Textile triboelectric nanogenerators simultaneously harvesting multiple "high-entropy" kinetic energies. *ACS Appl Mater Inter* 2021; **13**: 20145-52.
18. Nie S, Guo H and Lu Y *et al.* Superhydrophobic cellulose paper-based triboelectric nanogenerator for water drop energy harvesting. *Adv Materi Technol* 2020; **5**: 2000454.
19. Dishon Ben Ami S, Ehre D and Ushakov A *et al.* Engineering of pyroelectric crystals decoupled from piezoelectricity as illustrated by doped alpha-glycine. *Angew Chem Int Ed* 2022; **61**: e202213955.
20. Xu X, Wang T and Chen P *et al.* Femtosecond laser writing of lithium niobate ferroelectric nanodomains. *Nature* 2022; **609**: 496-501.
21. Jiang J, Zhang L and Ming C *et al.* Giant pyroelectricity in nanomembranes. *Nature* 2022; **607**: 480-5.
22. Lheritier P, Torello A and Usui T *et al.* Large harvested energy with non-linear pyroelectric modules. *Nature* 2022; **609**: 718-21.
23. Zhou Y, Ding T and Gao M *et al.* Controlled heterogeneous water distribution and evaporation towards enhanced photothermal water-electricity-hydrogen production. *Nano Energy* 2020; **77**: 105102.
24. Kim J, Lee JH and Ryu H *et al.* High-performance piezoelectric, pyroelectric, and triboelectric nanogenerators based on P(VDF-TrFE) with controlled crystallinity and dipole

alignment. *Adv Funct Mater* 2017; **27**: 1700702.

25. Jachalke S, Mehner E and Stöcker H *et al.* How to measure the pyroelectric coefficient? *Appl Phys Rev* 2017; **4**: 021303.
26. Yang J, Chen Q and Xu F *et al.* Epitaxy enhancement of piezoelectric properties in P(VDF-TrFE) copolymer films and applications in sensing and energy harvesting. *Adv Electron Mater* 2020; **6**: 2000578.
27. Pegasusweb Mirko Cecchini. *World climate guide*. https://www.climatestotravel.com/2022 (1 March 2023, date last accesssed).
28. Liu X, Zhang X and Min J. Maximum spreading of droplets impacting spherical surfaces. *Phys Fluids* 2019; **31**: 092102.
29. Durey G, Magdelaine Q and Casiulis M *et al.* Droplets impaling on a cone. *Phys Rev Fluids* 2020; **5**: 110507.
30. Wang X-Q, Tan CF and Chan KH *et al.* Nanophotonic-engineered photothermal harnessing for waste heat management and pyroelectric generation. *ACS Nano* 2017; **11**: 10568-74.
31. Park T, Na J and Kim B *et al.* Photothermally activated pyroelectric polymer films for harvesting of solar heat with a hybrid energy cell structure. *ACS Nano* 2015; **9**: 11830-9.
32. Zabek D, Taylor J and Boulbar EL *et al.* Micropatterning of flexible and free standing polyvinylidene difluoride (PVDF) films for enhanced pyroelectric energy transformation. *Adv Energ Mater* 2015; **5**: 1401891.
33. Zhao T, Jiang W and Liu H*, et al.* An infrared-driven flexible pyroelectric generator for non-contact energy harvester. *Nanoscale* 2016; **8**: 8111-7.
34. Ma N, Zhang K and Yang Y. Photovoltaic-pyroelectric coupled effect induced electricity for self-powered photodetector system. *Adv Mater* 2017; **29**: 1703694.
